# A novel approach for congenital absence of the uterine cervix: Office hysteroscopic versapoint canalization using real-time trans-abdominal sonography guidance

**DOI:** 10.4274/tjod.59219

**Published:** 2016-03-10

**Authors:** Bülent Haydardedeoğlu, Pınar Çağlar Aytaç

**Affiliations:** 1 Başkent University Faculty of Medicine, Department of Obstetrics and Gynecology, Division of Reproductive and Endocrinology Unit, Adana, Turkey

**Keywords:** Cervical agenesis, hysteroscopy, Versapoint

## Abstract

Herein, we report a novel technique for cervical agenesis via office hysteroscopy using Versapoint using real-time trans-abdominal sonography guidance. Fourteen days after the canalization procedure, a second hysteroscopy was performed to remove the silicone catheter and insert a Cupper T380a intrauterine device, which aimed to prevent a neocervical canal occlusion. This is the first case report of a patient with congenital cervical agenesis undergoing canalization with Versapoint in an office hysteroscopy; laparoscopy was not performed for assistance.

## INTRODUCTION

Congenital absence of uterine cervical agenesis is an extremely rare anomaly among all urogenital tract malformations and occurs in 1 in 80.000-100.000 births^(1)^. The first presenting symptom of cervical agenesis is primary amenorrhea with secondary sex characterstics, occasional cyclic lower abdominal pain, and commonly-occurring retrograde menstruation. A total abdominal hysterectomy remains the primary choice of treatment in these patients for relieving symptoms of retrograde menstruation and preventing concomitant endometriosis. However, reconstructive surgery for saving the uterus is performed by only a few expert surgeons worldwide. This skillful surgery has been reported in the current literature as a case series^(2,3,4,5,6)^. Recently, Kriplani et al.^(7)^ reported a new case series of laparoscopic-assisted uterovaginal anastomosis in 14 patients. Laparoscopy is usually performed to assist in reconstruction of the utero-vaginal pathway^(8)^.

Herein, we report a novel technique for cervical agenesis in an office hysteroscopy using Versapoint with real-time trans-abdominal sonography guidance. After reconstructing the cervical canal, a silicone catheter remained for 14 days and was replaced by a Cupper-T 380 a intrauterine device (IUD) to prevent neocervical canal obliteration following a second hysteroscopic re-evaluation. At the 9-month follow-up, the patient was relieved of severe abdominal pain and has regular menstruation. This is the first case report of a patient with congenital cervical agenesis in whom laparoscopy was not performed for assistance.

## CASE REPORT

A woman aged 21 years was referred to Adana Başkent University Faculty of Medicine, Department of Obstetrics and Gynecology, Division of Reproductive Endocrinology/IVF Unit and Endoscopic Surgery with primary amenorrhea and long-standing severe cyclic pelvic pain. The patient had been married for 3 years but was divorced due to infertility. She went laparotomy for reconstruction 3 years ago, but this was unsuccessful. She was referred to a university hospital 2 years ago for reconstruction of an utero-vaginal anastomosis and underwent a laparoscopy for canalization, which also failed. On examination, she had a blind-ending vagina and normal secondary sexual characteristics. A karyotype analysis and other hormonal parameters had been evaluated as normal (normal female, 46 XX) at a previous university hospital. She was accepted for the operation and was admitted for surgery to canalize the uterovaginal junction. Institutional review board approval was not obtained because the approval of the patient was obtained.

## SURGICAL TECHNIQUE

**Figure 1:**
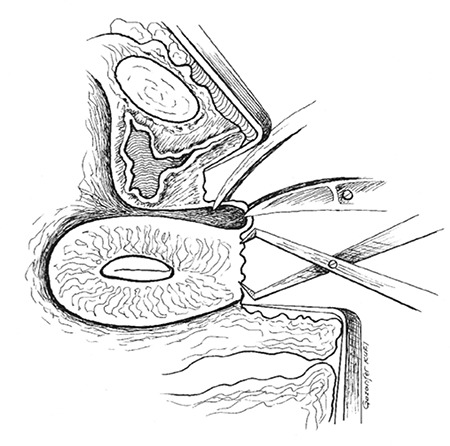
Dissection plane to create neocervix

**Figure 2:**
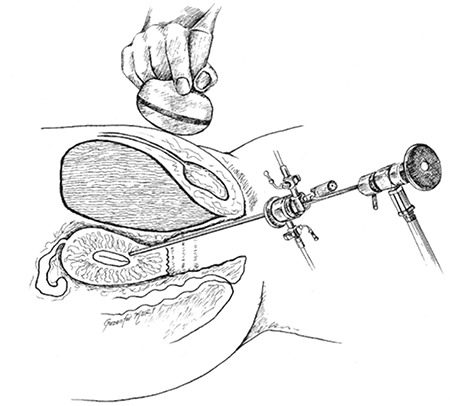
Hysterescopic instrumentation during uterine cavitiy access

After induction of general anesthesia and administration of an appropriate prophylactic antibiotic, the patient was prepared for surgery and placed in the supine position in the operating room. Her legs were placed in a lithotomy position at a 30° angle at the hips. The vulva, perineum, legs, and vagina were cleaned and draped. After inserting a speculum, suprapubic pressure was exerted to force the uterine body down. After palpation of the utero-vaginal junction, a transverse incision was made through the so-called vesico-cervical fascia, and the muscular layer of the bladder was removed upwards (Figure 1). The dense thick body of the lower uterine segment was visualized, and a 3 cm longitudinal incision (1 cm in depth) was made through the lower part of the uterine body (Figure 2). Thereafter, the bladder was filled using an office hysteroscope to visualize the trans-abdominal sonography.

**Figure 3:**
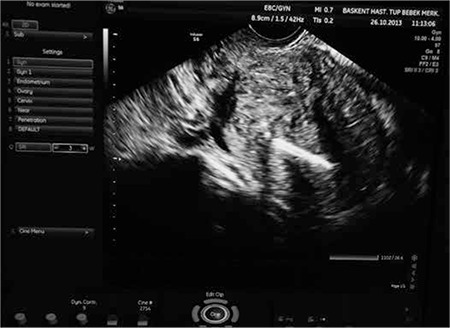
Ultrasonographic image of intrauterine device in the uterine cavity and the neocervix extending in the lower part of uterus

A 4 mm office hysteroscope (Karl Storz) was inserted into the previous linear incision with the assistance of gynecologic sonographer, and Versapoint cautery was performed to cut the blind end of the uterine body. The office hysteroscope was pushed forward with the cutting mode of the twizzle type Versapoint (Gynecare, Ethicon) with 0.9% NaCl distention medium through the ultrasonographically visible triple line (Figure 3). Hegar buji dilatators were used a few times to dilate the canal appropriately. After several attempts to reach the uterine fundus, the uterine cavity was visualized with the hysteroscope. Hegar buji dilatators were used to dilate the newly-formed canal. A silicone catheter was inserted into the uterine cavity and filled with 10 mL of saline to prevent obliteration of the canal. The vaginal mucosa was closed separately using 2/0 Vicryl.

Fourteen days after the canalization procedure, a second hysteroscopy was performed to remove the silicone catheter and insert a Cupper T380a IUD, which aimed to prevent a neocervical canal occlusion. An oral contraceptive (Yasmin, İstanbul, Turkey) was prescribed to the patient to schedule the first menstruation. The patient menstruated regularly for 4 days 25 days later. She was re-examined 10 days after ending the first menstruation; only a pinpoint neocervical orifice and the string of the IUD was visible (Figure 1, 2). The patient has had regular menstrual cycles at 27-day intervals and 4 days of bleeding since the first menstruation, without the long-standing pelvic pain or infection.

## DISCUSSION

Operative management of congenital uterine cervical agenesis is a challenging issue. With the advancement of endoscopic surgery skills, laparoscopic assistance has gained importance during the uterovaginal canalization procedure for cervical agenesis^(7,8)^. This extremely rare uterine anomaly has been corrected by several operative techniques in which earlier case series reported high recurrence and high complication and infection rates, particularly concomitant with vaginal agenesis^(9,10)^. Nguyen et al.^(6)^ used a vaginal mucosa-lined polytetrafluoroethylene graft to prevent closure of the reconstructed cervical canal; we used an IUD string and this was also effective.

This is the first case report of a Versapoint hysteroscopy without operating on the abdomen in terms of laparoscopy or laparotomy. Instead, real-time trans-abdominal ultrasound guidance was used to reach the correct route to the uterine cavity. Ultrasound guidance is less costly than laparoscopic guidance and adds no additional cost over hysteroscopy alone. In our clinic, ultrasonic guidance is routinely used throughout hysteroscopic synechiolysis for severe intrauterine adhesions. Some authors have also reported that ultrasound is the optimal choice for guidance during a difficult hysteroscopy^(11,12)^.

The Versapoint, which uses a 4 mm office hysteroscope, is a safe and effective alternative to the resectoscope. It is used predominantly in nulligravida women, particularly in those with cervical canal stenosis. The superiority of the office hysteroscope over the resectoscope in terms of smaller diameter made it the first choice in the present case. Only Versapoint could be used in such a narrow area. Furthermore, the superiority of Versapoint over resectoscopes during operative hysteroscopy has been shown by Litta et al.^(13)^.

Future fertility with this type of new technique is a challenging point. We do not know what will happen to the cervical canal after removal of the IUD. Cervical canal obliteration will be the main threatening complication after IUD removal. The obliteration of the newly-formed cervix can be corrected by repetitive hysteroscopy and a vaginal mucosa-lined polytetrafluoroethylene graft can be used if we face cervical canal obliteration^(6)^. In case of infertility, intrauterine insemination seems to be successful after this type of operation. However, transabdominal cerclage would be reasonable for preventing preterm labor in such patients.

The concomitancy of hematometra, hematosalpinx, endometriosis, and its complications would be seriously relieved after this type of operation. Our patient insisted on reporting dramatic relief of cyclic pain, which was due to correction of outflow obstruction. However, the addition of laparoscopic operation to congenital cervical agenesis can be superior for the evaluation of endometriosis staging. The advancement of endometriosis in such young patients is also controversial.

This novel approach is a safe, cheap, and feasible technique for congenital uterine cervical agenesis. The simplicity of this operation is in the presence of the blind-ending vagina. This technique is not suitable in cases of concomitant cervical and vaginal agenesis, in which laparoscopy should be used. The obstetric outcomes of such reconstructive surgery remain to be determined. Thus, further studies should evaluate the long-term gynecologic and obstetric outcomes following such an operation.
